# Crop rotation and the impact on soil carbon in the U.S. Corn Belt

**DOI:** 10.1186/s13021-025-00293-5

**Published:** 2025-04-26

**Authors:** Yining Wu, Eric C. Davis, Brent L. Sohngen

**Affiliations:** 1 Department of Agricultural Environmental, and Development Economics, The Ohio State University, Columbus, OH 43210 USA; 2 United States Department of Agriculture-Economic Research Service, Kansas City, MO 64105 USA

**Keywords:** Carbon sequestration, Soil organic carbon, Crop rotation, Corn Belt, Crop type, Corn and soybean

## Abstract

Soils are receiving increasing attention as carbon sinks that can reduce atmospheric CO_2_. While common Best Management Practices (BMP), such as cover crops, reduced or minimum tillage, and advanced nutrient management, have been considered as alternatives to build soil carbon storage in managed crop fields, crop-species choices have often been overlooked. This paper uses the Rapid Carbon Assessment (RaCA) data from U.S. Department of Agriculture (USDA), to examine how the rotation of two of the most widely used crops in the U.S., corn and soybeans, influences Soil Organic Carbon (SOC) stocks. We show that at the depths of 0 to 100 cm, corn is correlated with a higher level of SOC stocks than soybeans, and the more years that corn is cultivated the higher the SOC stocks. Specifically, an additional year of corn planted every 3 years is estimated to increase SOC stocks at depths of 0 to 100 cm by 25.1%. Based on our analysis, were all the land in the U.S. states of Ohio, Indiana, Iowa, and Illinois that are currently either mono-cropped with soybeans or follow some sort of soybean-corn rotation converted to corn mono-cropping, the estimated gain in SOC would be 896.7 million Mg C (1 Megagram = 1 ton). This represents a theoretical upper limit for SOC improvements. If current rotational practices were shifted such that corn was planted in 2 of every 3 years in the same region, the theoretical increase in SOC stocks is estimated to be 172.9 million Mg C. Multiplying this result by a Social Cost of Carbon priced at $678/t C in 2020 U.S. dollars (Rennert et al. in Nature 610:687–692, 2022), the total benefits are estimated at $117 billion.

## Introduction

According to the Intergovernmental Panel on Climate Change [19], under current emission trends of CO₂ and other greenhouse gas emissions, there is the potential for global warming thresholds of 1.5°C and 2°C to be exceeded in the coming decades occur. Soil Organic Carbon (SOC) is receiving increasing attention, as soils can be natural sinks for carbon, removing hard to abate emissions from the atmosphere [9, 18, 22, 29, 36]. SOC refers to the carbon component of organic compounds in soil [32]. SOC stocks increase with the addition of carbon inputs, mainly in the form of dead plant material or manure. Soils, however, may also be potential sources of CO₂ emissions through land use change and traditional crop production practices [6, 18], mainly caused by decomposition, leaching, and erosion [26]. From 1850 to 2015, U.S. agricultural land expanded by 110%, which has raised concern about the depletion of SOC stocks compared to the native ecosystems from which they were derived [3, 24].

Given the potential of soils and the large area of agricultural land, considerable effort has been devoted to finding and testing “best management practices” (BMP) for building SOC storage in managed crop fields, through practices such as cover cropping, reduced or minimum tillage, advanced nutrient management, and integrated crop-livestock systems [5, 7, 25]. Less attention has been paid to the decision over which crop to plant and how that choice affects SOC storage. There is evidence that perennials, including biomass energy crops or permanent cover, can lead to more SOC storage in soils than annual crops [2, 8, 37]. However, such proposals face headwinds due to their likely impact on crop outputs, market prices, and the financial well-being of farmers. This paper, therefore, considers whether two of the most widely used crops in the U.S. can be planted in a different combination to increase SOC stocks while still being supportive of food production.

Corn (*Zea mays* L.) and soybeans [*Glycine max* (L.) Merr.], the backbone of Midwestern U.S. crop production, are often grown in rotation to enhance yields and reduce input requirements [20, 21, 28]. For example, there is evidence in Iowa that if soybeans are grown in the prior year, corn yields will, on average, increase by 16.5 bu./ac. (a 13% increase from the baseline corn yield of 129.29 bu./ac.), and 51 lb./ac. of nitrogen (a 32% reduction from the typical application level of 160 lb./ac.) will be saved [13]. Likewise, if corn is grown in the prior year, soybean yield increases by 7 bu./ac. (25% from the baseline soybean yield of 28.04 bu./ac) [13].

Apart from the benefits in terms of yield and input costs, rotation complexity is also generally perceived to help increase soil health [40]. However, long-term field experiments have found that corn-soybean rotation systems have lower SOC storage compared to continuous corn systems [15, 27, 34]. The main explanation for this phenomenon is that the rotation of N-rich soybean litter and relatively N-poor corn litter may stimulate the decomposition of litter and SOC by promoting microbial growth after the corn phase and stimulating priming after the soybean phase [11, 17, 31].

To estimate the potential effects  of different crops on SOC stocks, researchers commonly utilize one of four approaches. The first approach is to use process-based models, like the Rothamsted Carbon Model (RothC) [23], DayCent [4], or the Agricultural Production Systems Simulator (APSIM) [22]. These models consider biogeochemical processes and are formulated according to mathematical-ecological theory. They can simulate SOC turnover and tie the site conditions to specific management practices but come with inherent limitations and challenges tied to data requirements, model parameterization, and uncertainties in model structure. Another alternative is to do field experiments [5, 14, 34], but these experiments might take years to detect changes in soil carbon pools. Moreover, measurements may be uncertain due to temporal and spatial variability. Other studies, therefore, have chosen the approach of conducting meta-analyses and compiling the results from existing available studies on the effect of management practices, such as cover crops and fertilizer management, on SOC stocks [12, 26]. However, these meta-analyses often struggle due to differing approaches to calculating SOC and variation in sampling methods across the databases [1, 10]. Given the limitations of the aforementioned methods, several studies to date have turned towards using publicly available nationwide databases (e.g., Soil Survey Geographic (SSURGO) data from the U.S. Department of Agriculture Natural Resources Conservation Service (USDA-NRCS), Rapid Carbon Assessment (RaCA) data from the USDA-NRCS, and Forest Inventory and Analysis (FIA) data from the USDA Forest Service) to evaluate regional carbon stocks [10, 41, 42].

Crop rotation sequences have not received significant attention in the literature outside of recommendations focused on shifting land to perennial crops or reducing the intensity of tillage. In this paper, we link the RaCA data [39] with the Cropland Data Layer (CDL) [38] of the United States Department of Agriculture—National Agriculture Statistics Service (USDA-NASS) to estimate the impact of common rotation sequences of corn and soybeans on SOC stocks in the Corn Belt. This study is one of the first to combine SOC data that was collected using a uniform approach across the U.S. with crop-specific land cover data. As the land cover data was collected using resolution satellite imagery, issues of data quality and consistency may be lessened compared to field experiments, and parameter uncertainty and model specification uncertainty are potentially reduced compared to process-based models.

## Materials and methods

Our estimation of the impact of crop type decisions on SOC stocks is derived from data from multiple sources. We use the RaCA data [39] to obtain contemporaneous measurements of SOC stocks and soil characteristics at depths of 5 cm, 30 cm, and 100 cm at 2,105 cropland sites across the United States. This cross-sectional data was collected by USDA-NRCS in 2010 and 2011 through a multi-level stratified random sampling scheme that used major land resource areas (MLRA) and a combination of soil groups and land use/land cover classes [39]. Next, using the latitude and longitude information of each RaCA site, we extract the crop types in the year of the SOC stock measurements from the Crop Data Layer (CDL) dataset [38]. CDL is an annual raster, geo-referenced, crop-specific land cover data layer with data from 2008 onwards with a ground resolution of 30 or 56 m depending on the state and year. We repeat the extraction process to add to our data the type of crops that were planted one and two years before the SOC stocks measurement. We also use data from the PRISM Climate Data [30] to understand the impact of temperature and precipitation. Unfortunately, our data did not allow us to fully control for all the inputs and management practices across different crops, and thus we consider the aggregate effects of crop species on SOC stocks and make the simplifying assumption that management is randomly distributed across crop type and location.

**Fig. 1:**
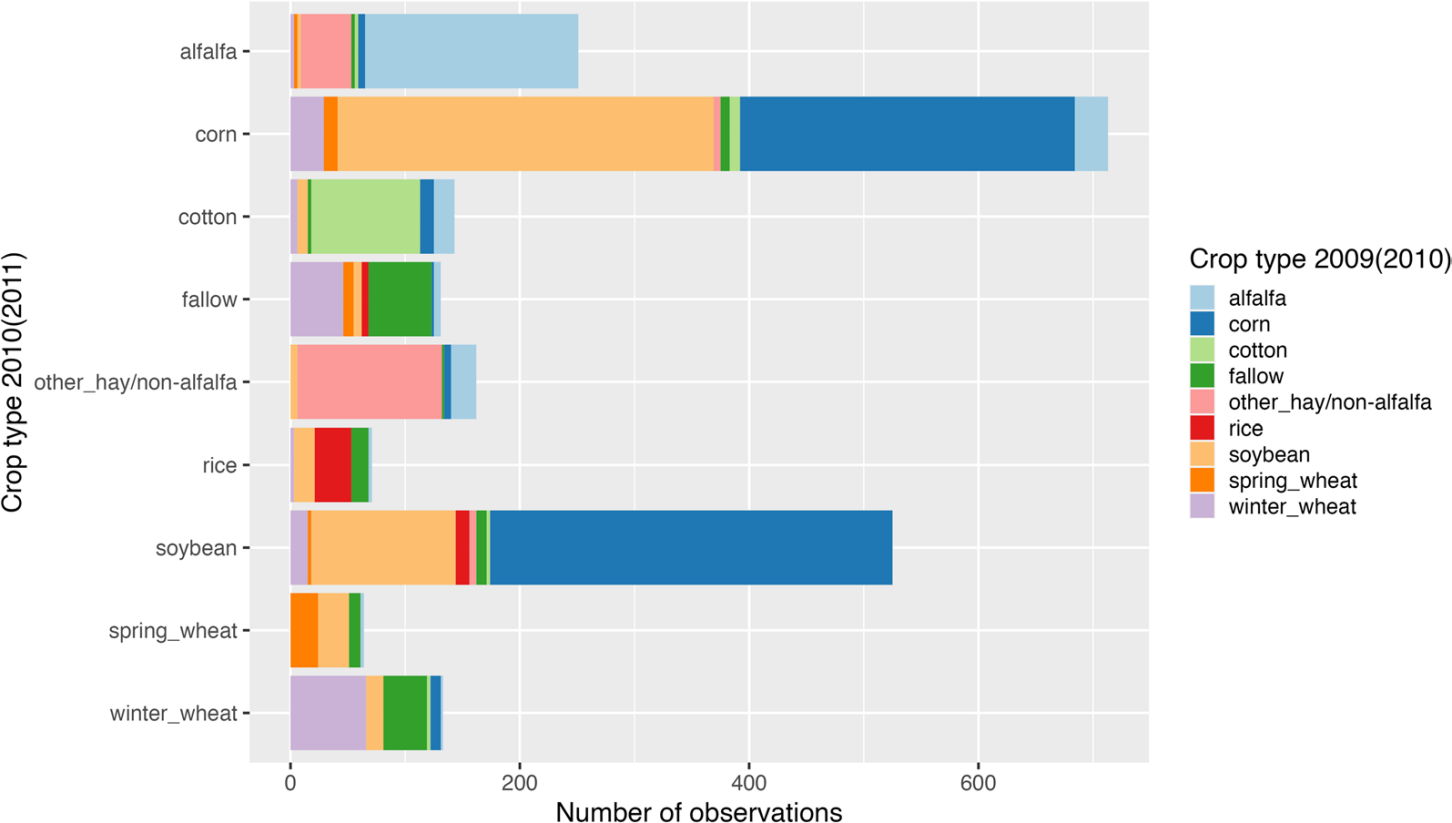
Distribution of 9 main crop types in 2010–2011 across crop types planted in 2009–2010 in RaCA

Looking at the distribution of crop types over the years SOC stocks were measured (2010 & 2011) and 1 year before the SOC stocks were measured (2009 & 2010), we find that the corn-soybean rotation is the predominant cropping system in the United States (Fig. 1). For corn planted in any given year, soybeans are the most likely crop planted in the previous year, followed by corn, winter wheat, and alfalfa. For soybeans, corn is the most likely crop planted the previous year, followed by soybeans and winter wheat. These results illustrate that a large share of the cropland in the United States is managed with an annual corn-soybean rotation. Another relatively large share experiences corn or soybeans being repeated for one or two years, but there is a relatively modest area of continuous corn or soybeans. Because of the predominance of these two crops, we focus our analysis on corn and soybeans and their impact on SOC stocks.

**Fig. 2:**
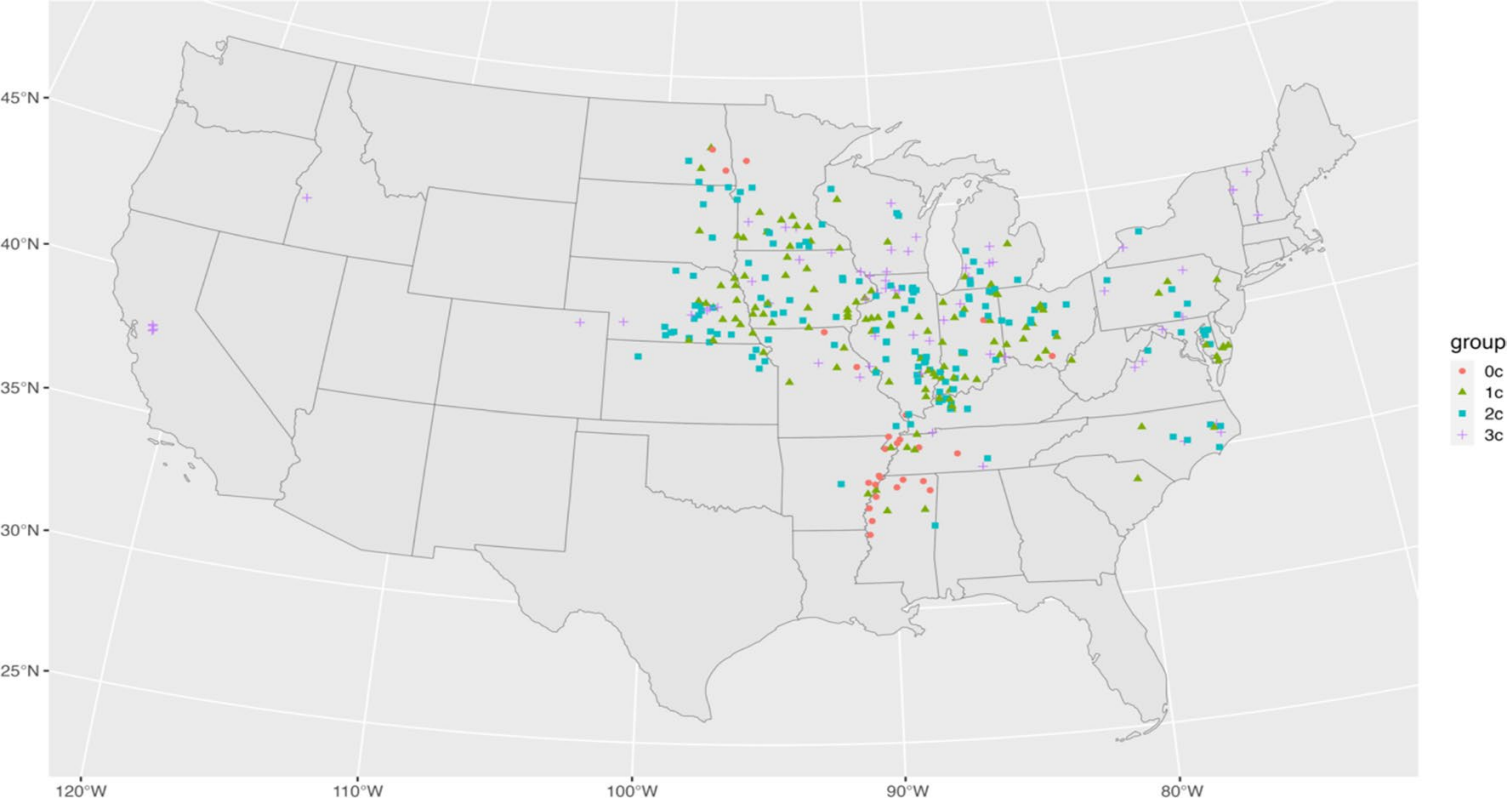
Spatial distribution of the corn-soybean rotation observations in RaCA

As Fig. 2 shows, most of the RaCA observations of the corn-soybean rotation system are located in the U.S. Corn Belt, and in particular, the states of Ohio, Indiana, Iowa, and Illinois. Over the 3 years immediately preceding the measurement of SOC stocks, the most common rotation saw corn planted for 2 years and soybeans for 1 year. The next most prevalent rotation had corn planted for 1 year and soybeans for 2 years. Less common rotations were mono-cropping of corn and finally mono-cropping of soybeans. We thus divide our data into four groups based on the number of years of corn in the 3-year period from zero to three (labeled as 0c, 1c, 2c, and 3c).

**Fig. 3:**
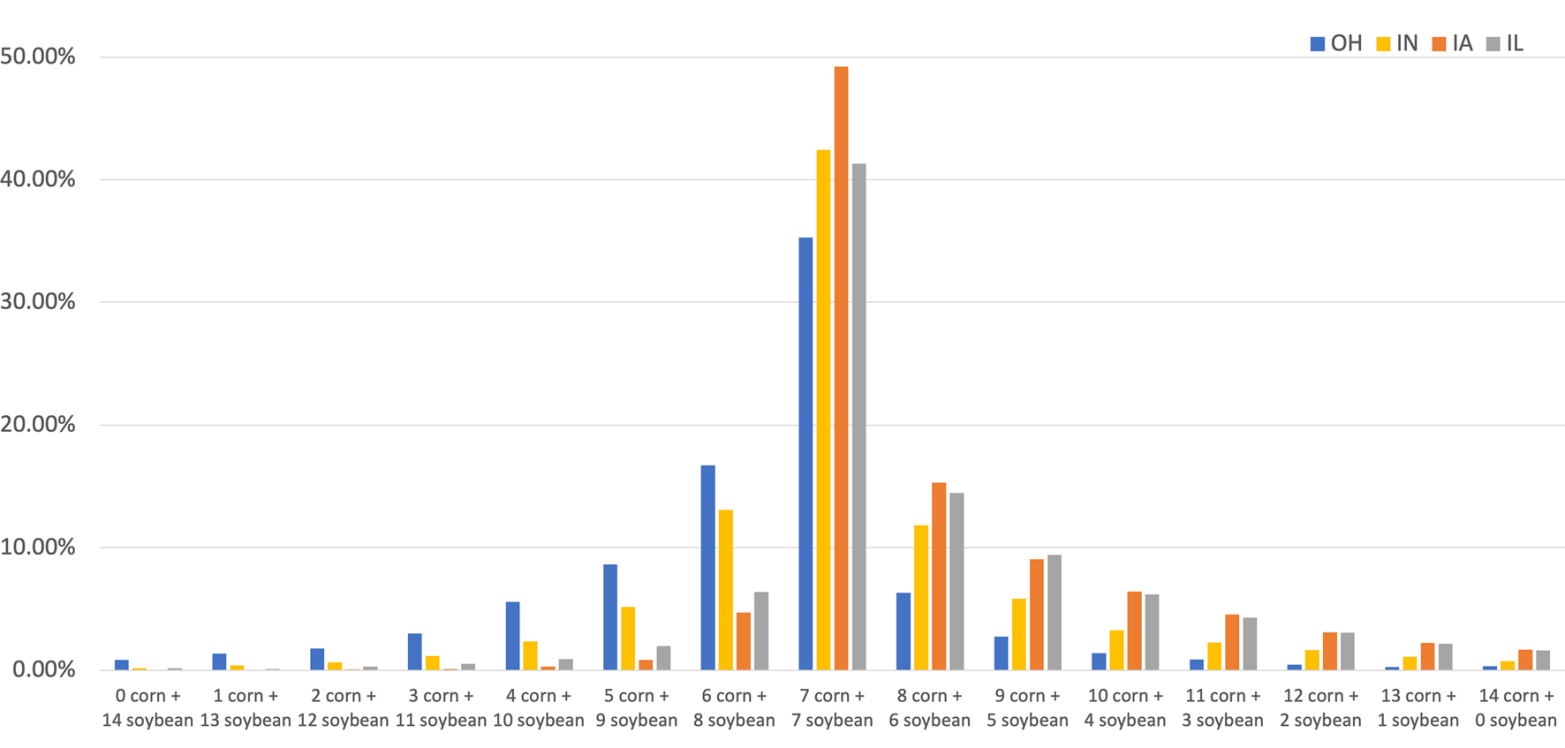
Average number of years that corn and soybeans were planted from 2008 to 2021 in the U.S. Corn Belt states of Ohio, Indiana, Iowa, and Illinois

To better understand the cropping decisions in these Corn Belt states, we randomly sample 40,000 points within the CDL in Ohio, Indiana, Iowa, and Illinois and examine the most common corn-soybean sequences over the 14-year period from 2008 to 2021. The sampling is performed using the sample() function in R, which generates random selections of 40,000 points within each state from the set of valid (non-NA) CDL raster grid cells after masking out areas outside state boundaries. This approach yields a statistically representative sample of crop cover values and their corresponding spatial locations within each state's agricultural land. Across all four states, the most common practice appears to have been equal planting of corn and soybeans (Fig. 3). In Iowa and Illinois, however, corn was planted more often than soybeans. In contrast, Ohio and Indiana farmers were more likely to plant soybeans. These differences are most likely the result of differences in soils and climate. Ohio and Indiana have a greater preponderance of heavier, clay soils, while sandy soils become more prevalent further west. Precipitation is also higher further east, especially in winter and early spring, which could influence planting decisions for farmers.

To understand SOC stocks as a function of cropping choices, we model the SOC stocks at depths of 0 to 100 cm at each site. We use the natural logarithm of the SOC stock values in the analysis. Control variables include environmental characteristics, including past weather (temperature and precipitation), latitude, and 16 USDA soil texture classification dummy variables. The classifications, determined during field samples, are based on the particle size distribution (proportions of sand, silt, and clay). For the weather variables, we trace back to 3 years before the year the SOC stocks were measured and create variables for the recent weather (12 months preceding the measurement) and the historical weather (13–36 months preceding the measurement). We further divide the data into warm months (April-September) and cold months (October–March) to prevent the impact of extreme hot and extreme cold weather from being obscured through the calculation of mean values. We also include area and month fixed effects. A summary of the descriptive statistics is shown in Table A1.

The regression equation we estimate is given as:

1 $$\begin{gathered} log\left( {SOC_{i} } \right) = \beta_{0} + \beta_{1} Crop_{0i} + \beta_{2} Crop_{1i} + \beta_{3} Crop_{2i} \hfill \\ + \beta_{4} Lat_{i} + \beta_{5} Weather_{i} + \beta_{6} Soil texture_{i} + \beta_{7} X_{i} + \varepsilon_{i} \hfill \\ \end{gathered}$$

where *i* indexes site. $SO{C}_{i}$ denotes the Soil Organic Carbon stock (Mg/ha C, 1 Megagram = 1 ton) for site *i*. ${Cro{p}_{0}}_{i}$, ${Cro{p}_{1}}_{i}$, and ${Cro{p}_{2}}_{i}$ indicate the crop type (corn or soybeans) in the year the SOC stocks were measured, the year before the year the SOC stocks were measured, and 2 years before the year the SOC stocks were measured. ${Lat}_{i}$ is the latitude of site *i*. ${Weather}_{i}$ includes 8 variables—mean temperature (precipitation) in the most recent 6 warm months, mean temperature (precipitation) in the warm months that fall between the 7th and 18th month prior to the measurement, mean temperature (precipitation) in the most recent 6 cold months, and mean temperature (precipitation) in the cold months that fall between the 7th and 18th month prior to the measurement. ${Soil texture}_{i}$ is a control that captures the impact of soil texture at site *i*. ${X}_{i}$ represents the area and month fixed effects. ${\varepsilon }_{i}$ is error term.

Considering that the crop types in the 3 years can be correlated, we then modify Eq. 1 into:

2 $$\begin{array}{c}log\left(SO{C}_{i}\right)={\beta }_{0 }{+ {\beta }_{1}Rotatio{n}_{i} +{\beta }_{2}{Lat}_{i} + \beta }_{3}{Weather}_{i}+{\beta }_{4}{Soil texture}_{i}+{\beta }_{5}{X}_{i}+{\varepsilon }_{i}\end{array}$$

where $Rotatio{n}_{i}$ indicates the number of years corn is cultivated in the three-year cropping system (i.e., 0c, 1c, 2c, and 3c).

**Fig. 4:**
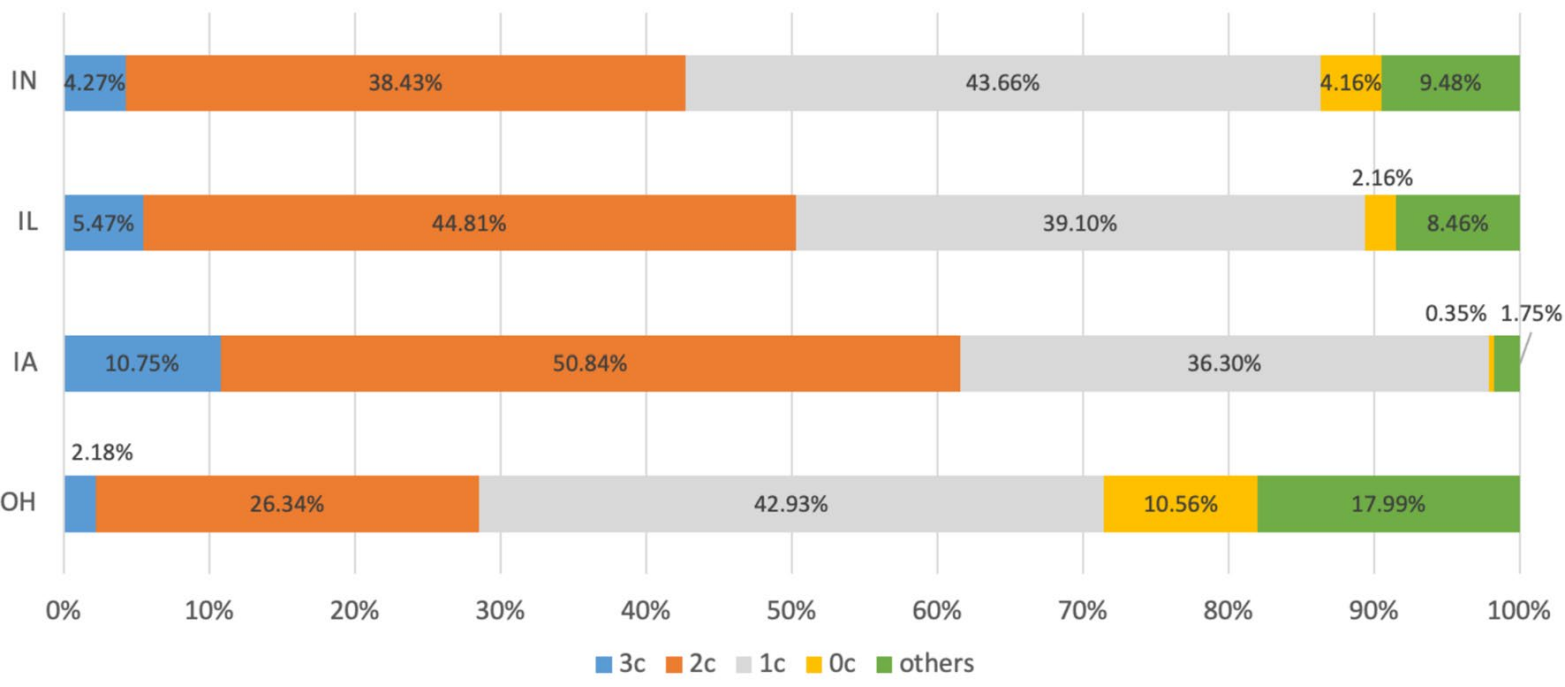
Proportion of total corn-soybean area, by number of years that corn was planted, in the U.S. Corn Belt states of Ohio, Indiana, Iowa, and Illinois, 2019–2021

When we attempt to gain an understanding of the potential of the U.S. Corn Belt to increase SOC stocks through changes in current crop rotation practices, we use the 40,000 points within the CDL for Ohio, Indiana, Iowa, and Illinois that had previously been sampled to quantify the proportion of land that follows each of the major rotational patterns (0c, 1c, 2c, and 3c) from 2019 to 2021. We find that the relative percentage of soybean mono-cropping (0c) and 1-year planting of corn (1c) is higher in Ohio and Indiana than in Illinois and Iowa (Fig. 4). Specifically, 10.56% of total land area was covered by soybean mono-cropping during the 3 years in Ohio, with 4.16% in Indiana, 2.16% in Illinois, and 0.35% in Iowa. 10.75% of total land area was covered by mono-cropping of corn during the 3 years in Iowa, followed by 5.47% in Illinois, 4.27% in Indiana, and 2.18% in Ohio.

## Results

**Fig. 5:**
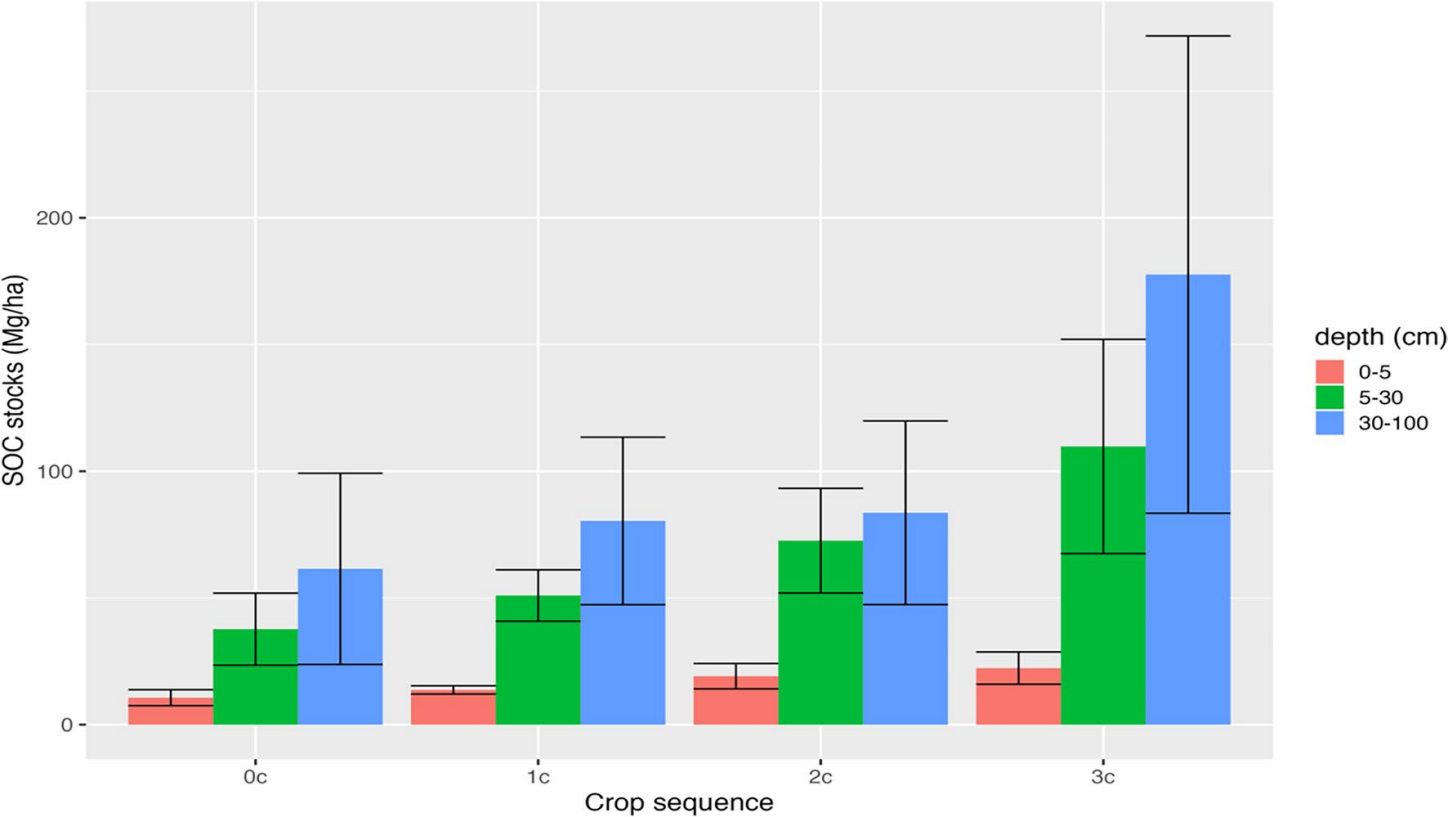
SOC stocks (Mg/ha C)  based on number of years corn is planted in corn-soybean systems, at depths of 0-5 cm, 5-30 cm, and 30-100 cm (95% CI)

SOC stocks exhibit significant variance among different crop sequences in the corn-soybean cropping system as Fig. 5 shows. Across all sequences, the deep layer (30–100 cm) contains more SOC than both the middle layer (5–30 cm) and shallow layer (0–5 cm). Soils with more years of corn planted in the 3 years immediately preceding the measurement of SOC stocks also contain higher SOC stocks in each layer. Altogether, when summing the SOC stocks at all depths from 0–100 cm, soybean-mono-cropping soils (0c) have the lowest mean SOC stocks (109.73 Mg/ha C) and corn-mono-cropping soils (3c) have the highest mean SOC stocks (300.20 Mg/ha C).

To better identify whether SOC stocks increased along with the number of years that corn was planted, we perform t-tests for the difference of log [SOC stocks (0-100 cm) (Mg/ha C)] between different corn-soybean rotations. Due to the unequal population variances, we employ one-tailed Welch two-sample t-tests. Table 1 shows that relative to soybean mono-cropping, 1-year corn rotations have greater SOC stocks with a confidence level of *p* < 0.10, while 2 years and 3 years of corn have higher confidence levels (*p* < 0.05 and *p* < 0.01, respectively). Relative to 1-year corn rotations, 2-year corn rotations have greater SOC stocks with a confidence level of *p* < 0.10, and 3-year corn rotations have greater SOC stocks with a confidence level of *p* < 0.01. Finally, soils under mono-cropping of corn relative to soils under 2-year corn rotations contain higher SOC stocks with a confidence level of *p* < 0.05.

**Table 1:** One-tailed Welch Two Sample t-test for difference of log(SOC stocks (0-100 cm) (Mg/ha C)), by number of years corn planted in corn-soybean systems (95%)

Null Hypothesis (H0)	Estimate	Statistic	P.value	Conf.low	Conf.high
log(SOC(1c))-log(SOC(0c)) < 0	0.239	1.41	0.0845	− 0.0487	Inf
log(SOC(2c))-log(SOC(0c)) < 0	0.386	2.27	0.0149	0.0983	Inf
log(SOC(3c))-log(SOC(0c)) < 0	0.671	3.17	0.00121	0.317	Inf
log(SOC(2c))-log(SOC(1c)) < 0	0.147	1.52	0.0646	− 0.0125	Inf
log(SOC(3c))-log(SOC(1c)) < 0	0.432	2.71	0.00408	0.167	Inf
log(SOC(3c))-log(SOC(2c)) < 0	0.285	1.79	0.0386	0.02	Inf

As corn appears to positively influence SOC stocks, Table 2 examines whether that influence differs depending on when corn was planted. Results suggest that recency matters. That is, at depths of 0–5 cm, 30–100 cm, and 0–100 cm, soils planted with corn in the SOC stock measurement year had a greater increase in SOC than those planted with soybeans in the measurement year and corn in the previous year. At depths of 5–30 cm, previous year planting of corn was shown to have a larger impact. Results suggest that having corn planted in either of the two years before the measurement increased SOC stocks relative to soybean mono-cropping by 12.0% to 27.6% (*p* < 0.05). Were corn to be planted in the first year and followed with 2 years of soybeans, results show no significant increase in SOC stocks.

**Table 2:**
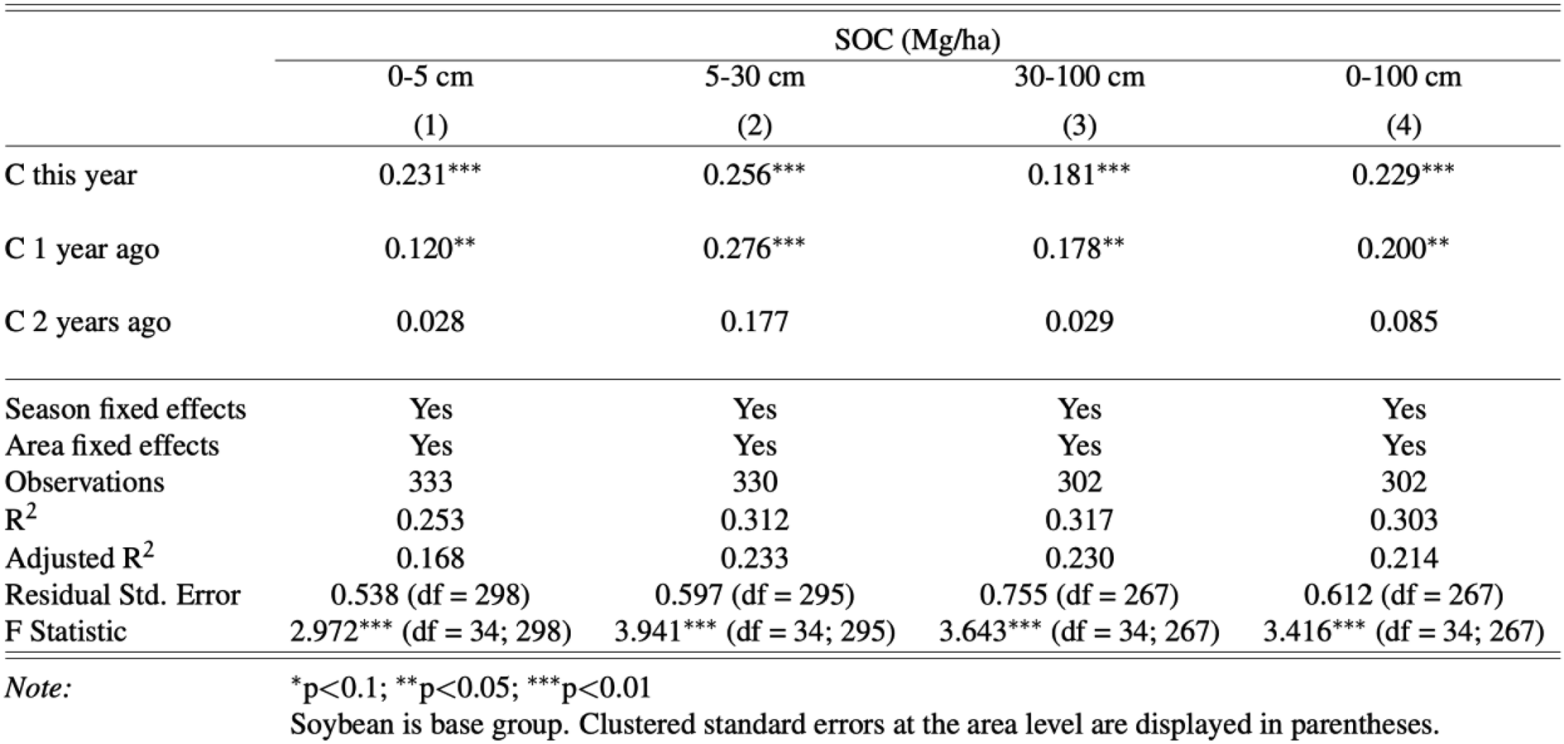
The effects of planting corn or soybeans on SOC(Mg/ha C) stocks in corn-soybean rotations

Next, we estimate a model that examines how SOC stocks vary by depth in relation not to the recency of corn being planted but to the number of years that corn was planted in the 3 years immediately preceding the measurement of SOC stocks. The results in Table 3 show that soils under more years of corn cultivation have higher SOC stocks across the depths of 0–5 cm, 5–30 cm, and 30–100 cm. Overall, at depths from 0 to 100 cm, compared to soybean mono-cropping soils, soils with corn planted in 1 of the 3 years preceding the SOC stock measurements had 30.0% more SOC (*p* < 0.05), and soils with corn planted in 2 of the 3 years preceding the measurements of SOC stocks had 42.4% more SOC (*p* < 0.10). Finally, corn mono-cropping soils had 71.3% more SOC compared to soybean mono-cropping soils (*p* < 0.01).

**Table 3:**
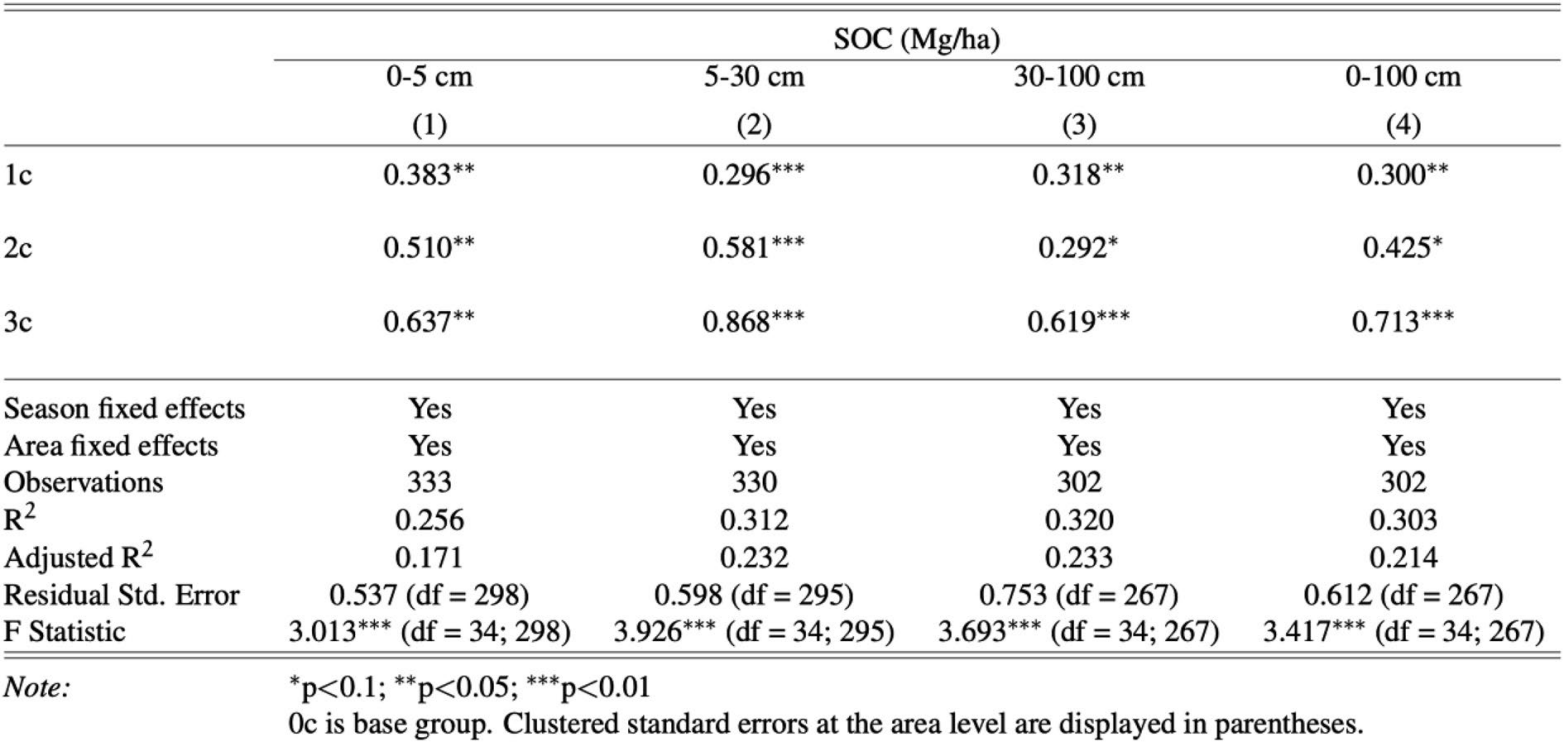
The effects that the number of years corn is planted has on SOC(Mg/ha C) stocks in corn-soybean rotations

**Fig. 6:**
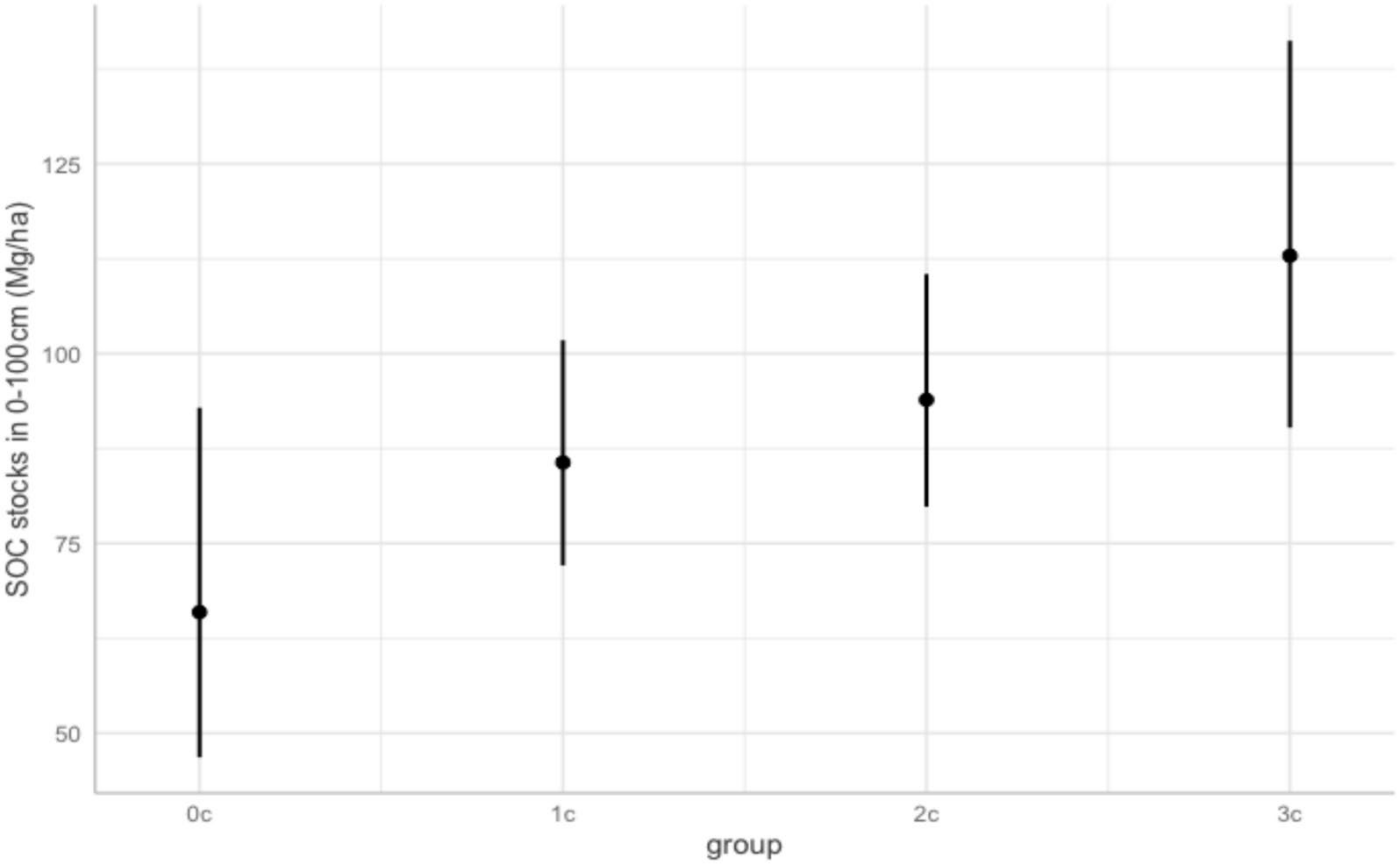
Marginal effect of groups on SOC(Mg/ha C) stocks at depths of 0–100 cm (95% CI)

We next quantify the gain in SOC stocks, on average, at depths from 0–100 cm based on the number of years that corn was cultivated in the 3 years immediately preceding the SOC stock measurements. Column 1 of Table 4 shows an increase in SOC compared to soybean mono-cropping and the difference grows with each additional year that corn is planted. For example, corn mono-cropping engenders 71.3% more SOC than soybean mono-cropping (*p* < 0.01). Column 2 shows the comparison using 1 year of corn planting as the base and shows similar results: corn mono-cropping increases SOC by 31.8% relative to soybean mono-cropping (*p* < 0.01). Columns 3 and 4 confirm those effects. The marginal effects are shown in Fig. 6. Robustness check results shown in Tables A5 and A7 show similar results with full crop sequences and across different model specifications.

**Table 4:**
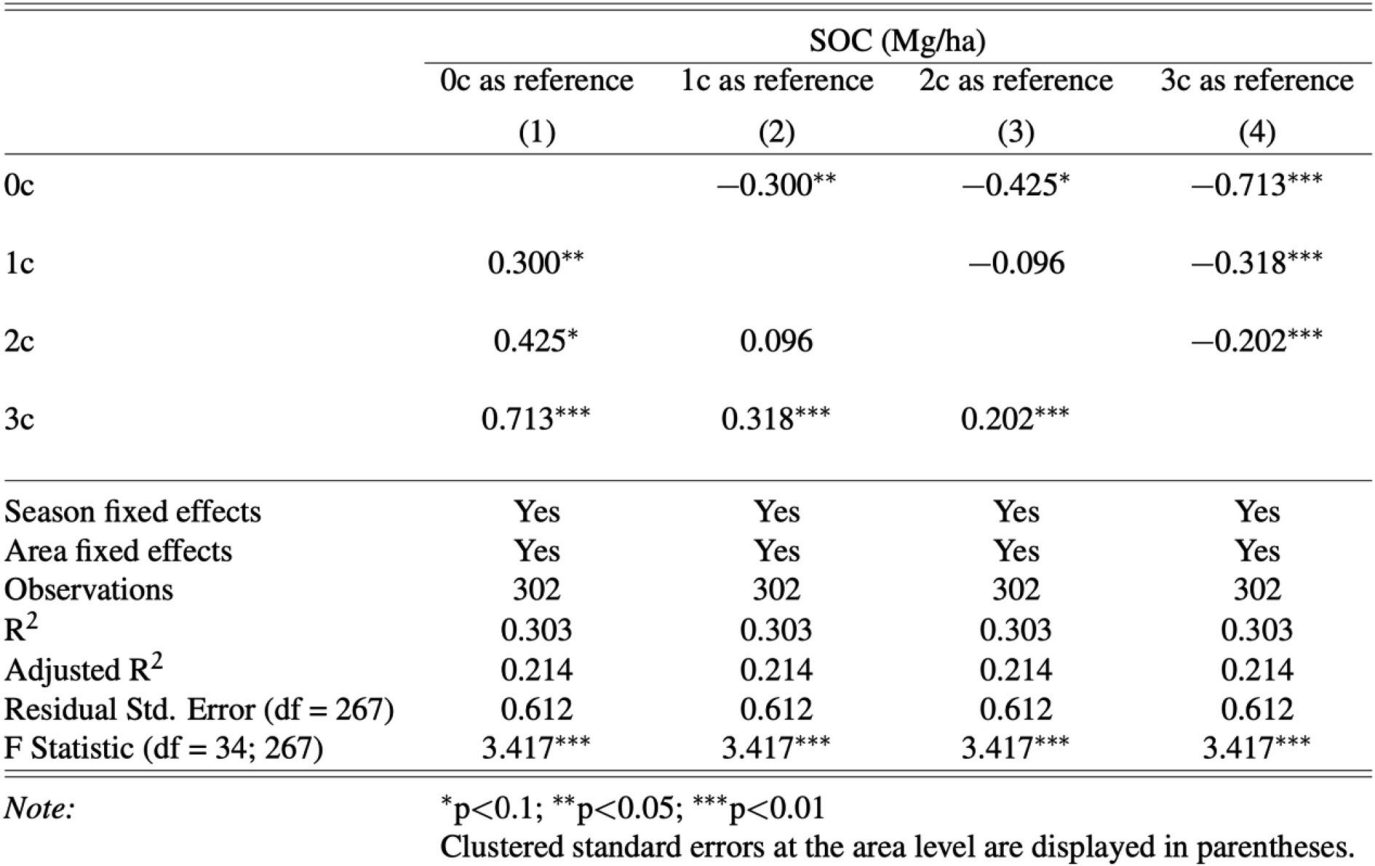
The effects that the number of years corn is planted has on SOC(Mg/ha C) stocks in corn-soybean rotations, using different base groups

### Scenario analysis

Our results indicate that SOC stocks at depths from 0 to 100 cm are higher when corn is planted than when soybeans are planted, with corn mono-cropping having considerably higher SOC stocks relative to soybean mono-cropping. It is possible that if policymakers want to increase SOC stocks on agricultural lands, they could incentivize more corn production. However, corn mono-cropping is likely to be costly in many locations and may not even be feasible agronomically. Even adding additional corn into the crop rotation may have financial implications in terms of input costs and crop yields. While understanding that there would be drawbacks, we aim to quantify the potential carbon change that would be associated with adding additional years in corn into crop rotations in the states of Ohio, Indiana, Illinois, and Iowa.

This analysis results in six hypothetical scenarios using our calculations of crop-choice patterns for each state (Fig. 4) and our regression results (Table 4). The first three scenarios assess the impact of adding corn to fields that data suggests are maintained only in soybeans. In the three scenarios we consider 1 year of corn with 2 years of soybeans, 2 years of corn with 1 year of soybeans, and 3 years of corn, all relative to soybean monocropping. The fourth and fifth scenarios examine the gains associated with adding 1 additional year of corn or 2 additional years of corn to a field that currently sees corn planted once every three years. Finally, we examine the potential gains from converting fields traditionally planted with corn twice every three years to fields where corn is mono-cropped. For each of these scenarios, we estimate the aggregate increase in SOC that would result from these cropping decisions.

Table 5 shows the area of each type of field and the potential increase in SOC stocks across the six hypothetical outcomes in the four states. First, due to the limited area of the land that is currently mono-cropped with soybeans, the potential increase of SOC stocks by adding 1 year, 2 years, and 3 years of corn are estimated to be just 24.3 million Mg C, 34.4 million Mg C, and 57.7 million Mg C, respectively. Importantly, each of these scenarios is mutually exclusive. That is, 57.7 million Mg C is the maximum potential increase in SOC storage possible for lands current cropped as soybeans only.

**Table 5:** Area of land in various crop rotations 2019–2021, and potential change in SOC stocks across policies and states

	OH	IA	IL	IN	Corn Belt
**Area of land (hectare)**					
Mono-cropped with soybeans out of 3 years	352,647	31,700	178,870	174,739	737,955
**Increase of SOC (Mg C)**					
by adding 1 year of corn	11,589,507	1,041,791	5,878,430	5,742,692	24,252,420
by adding 2 years of corn	16,438,138	1,477,639	8,337,753	8,145,227	34,398,758
by adding 3 years of corn	27,574,900	2,478,732	13,986,541	13,663,581	57,703,754
**Area of land (hectare)**					
Cropped with soybeans for 1 year and corn for 2 years out of 3 years	1,434,097	3,270,538	3,387,646	1,895,721	9,988,002
**Increase of SOC (Mg C)**					
by adding 1 year of corn	19,892,410	45,365,749	46,990,159	26,295,609	138,543,927
by adding 2 years of corn	65,578,921	149,556,379	154,911,542	86,688,221	456,735,063
**Area of land (hectare)**					
cropped with soybeans for 2 years and corn for 1 year out of 3 years	880,021	4,571,320	3,708,637	1,615,100	10,775,078
**Increase of SOC (Mg C)**					
by adding 1 year of corn	31,218,324	162,165,459	131,562,148	57,294,931	382,240,862

With much larger area, converting fields that are traditionally planted with corn for 1 year to corn for 2 years and mono-cropping is estimated to increase SOC stocks by 138.5 million Mg C and 456.7 million Mg C, respectively. Finally, converting fields that are traditionally planted with corn for 2 years to mono-cropping of corn are estimated to increase SOC stocks by 382.2 million Mg C.

Taken together, were all the land in the U.S. states of Ohio, Indiana, Iowa, and Illinois that are currently either mono-cropped with soybeans or follow some sort of soybean-corn rotation converted to corn mono-cropping, the estimated gain in SOC would be 896.7 million Mg C. Iowa's extensive cropland area makes it the leading contributor of these four states (314.2 million Mg C), followed by Illinois (300.5 million Mg C), Indiana (157.8 million Mg C), and Ohio (124.4 million Mg C).

## Discussion

Our analysis demonstrates that SOC stocks could be increased through modifications to corn-soybean rotation practices in the U.S. Corn Belt. While the theoretical maximum gain of 896.7 million Mg C through conversion to corn mono-cropping represents a substantial carbon sequestration opportunity, this scenario requires careful consideration of multiple environmental and economic tradeoffs.

It is worth noting that crop rotations have been widely shown as an effective approach for improving yields [28, 35]. Yields of rotated crops are higher because rotations reduce pest problems and enrich soils. Soybeans are commonly planted before corn to reduce fertilizer costs for corn [13, 21]. The increased nitrogen fertilizer application associated with intensified corn production has the potential to lead to increased nitrous oxide (N_2_O) emissions, a greenhouse gas with approximately 300 times the warming potential of CO_2_ over a 100-year period [19]. Additionally, the Corn Belt has been continuously experiencing significant water quality issues related to nitrogen loading in waterways from fertilizer [16].

Market effects present another important consideration. A large-scale shift toward more intensive corn rotation would likely impact grain markets substantially. Reduced soybean production may result in higher soybean prices, potentially affecting international trade patterns and food security. These market responses could induce land use changes elsewhere that might offset some of the carbon sequestration benefits achieved through increased SOC stocks in the Corn Belt.

Given these considerations, while our analysis suggests that increasing corn rotation intensity offers significant potential for soil carbon sequestration, there are numerous tradeoffs for decision makers to consider. Quantifying these various tradeoffs is a task we leave to the integrated assessment modeling community, as those tools seem well suited to capture both the direct and indirect effects of rotation changes.

## Conclusions

Crop rotation sequences have not received significant attention as avenues to increase SOC stocks, outside of recommendations focused on shifting land to perennial crops or reducing the intensity of tillage. By combining national SOC stocks assessment data and satellite-derived crop-specific land cover data, we find a significantly positive relationship between increased cultivation of corn and SOC stocks at depths of up to 100 cm. Specifically, an additional year of corn cultivation during any 3-year period is shown to increase SOC stocks, on average, by 25.08%.

Given this, we examine 6 hypothetical policies that would increase the frequency of corn being planted in the current corn-soybean rotations in the U.S. Corn Belt states of Ohio, Indiana, Iowa, and Illinois. If all the land in these states, which was currently either mono-cropped with soybeans or followed some sort of soybean-corn rotation, was converted to corn mono-cropping, the estimated gain in SOC would be 896.7 million Mg C. This essentially shows the theoretical upper bound for the potential increase of SOC stocks by switching crop types between corn and soybeans. Converting fields such that 2 years out of every 3 are planted with corn has the potential to increase SOC stocks by 172.9 million Mg C. While that figure is 723.8 million Mg C less than the gain obtained through conversion to corn mono-cropping, it nevertheless gives another indication of the estimated potential to increase carbon sequestration. If this result is multiplied by a mean Social Cost of Carbon estimate of $678/t C [33], the total benefits are estimated at over $117 billion. However, carbon sequestration is not the only societal goal, as food security, the financial health of farmers, and other environmental challenges are also important concerns.

To foster future research and a better understanding of soil dynamics, a movement beyond cross-sectional nationwide SOC datasets to panel data seems merited. Cross-sectional data is unable to well capture time dynamics or control for unobserved management heterogeneity. The development of a nationwide repeated-measured SOC panel dataset that was coupled with information on management practices at the point level could potentially aid the research community in rigorously evaluating the effectiveness of "BMPs" in enhancing SOC storage in the future.

## Figures and Tables

**Table 7:**
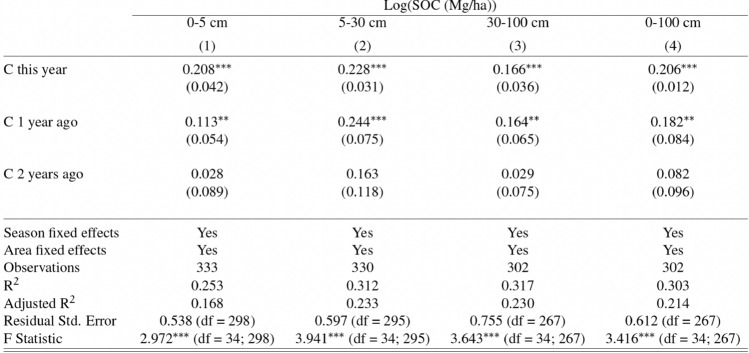
The effects of planting corn or soybean on log(SOC(Mg/ha C)) stocks in corn-soybean rotations (raw regression result)

Soybean is base group. Clustered standard errors at the area level are displayed in parantheses

*p<0.1

**p<0.05

***p<0.01

**Table 8:**
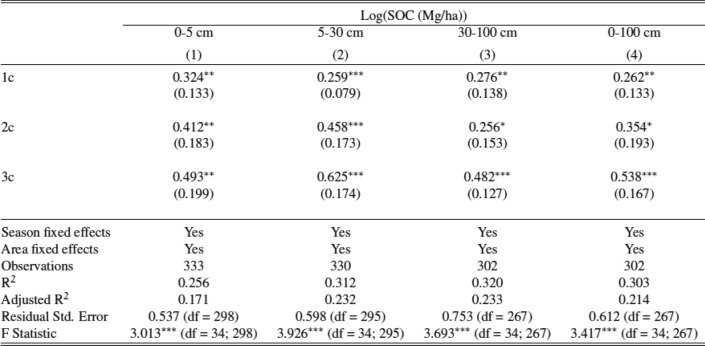
The effects of the years of corn plantation on log(SOC(Mg/ha C)) stocks in corn-soybean rotations (raw regression result)

0c is base group. Clustered standard errors at the area level arew displayed in parantheses

*p<0.1

**p<0.05

***p<0.01

**Table 9:**
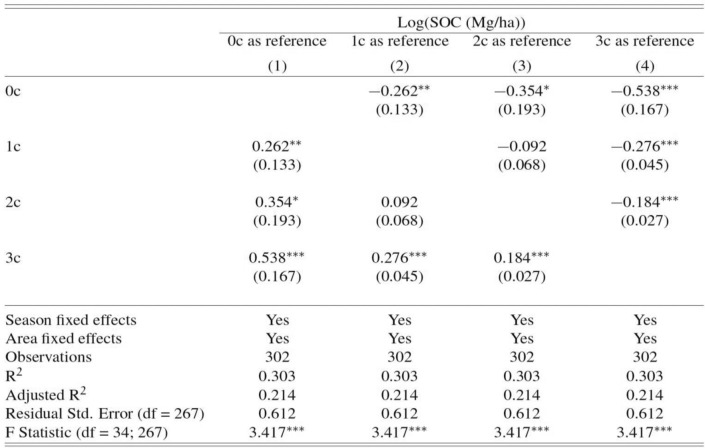
The effects of the years of corn plantation on log(SOC(Mg/ha C)) stocks in corn-soybean rotations with different base group (raw regression result)

Clustered standard errors at the area level arew displayed in parantheses

*p<0.1

**p<0.05

***p<0.01

**Table 10:**
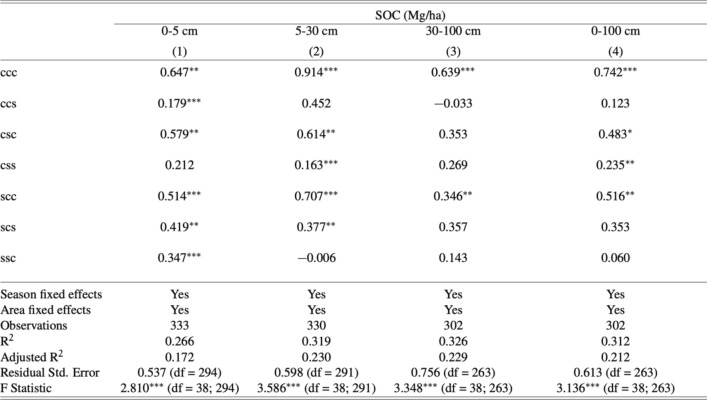
The effects of different sequences of corn-soybean rotation on SOC(Mg/ha C) stocks at different depths

c is short for corn and s is short for soybean. The sequences indicate crop type c/s 2 years before - c/s 1 year before - c/s the year of SOC stocks measurement. sss is base group. Clustered standard errors at the area level arew displayed in parantheses

*p<0.1

**p<0.05

***p<0.01

**Table 11:**
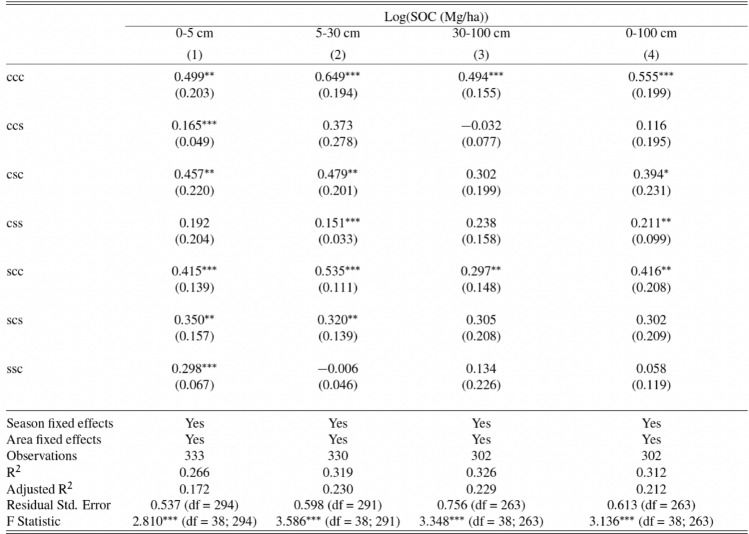
The effects of different sequences of corn-soybean rotation on log(SOC(Mg/ha C)) stocks at different depths (raw regression result)

c is short for corn and s is short for soybean. The sequences indicate crop type c/s 2 years before -c/s 1 year before- c/s the year of SOC stocks measurement. sss is base group. Clustered standard errors at the area level arew displayed in parantheses

*p<0.1

**p<0.05

***p<0.01

**Table 12:**
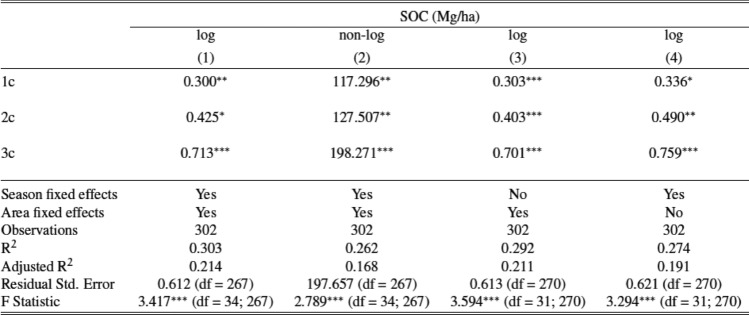
The effects of different sequences of corn-soybean rotation on SOC(Mg/ha C) stocks across model specifications

0c is base group. Clustered standard errors at the area level arew displayed in parantheses

*p<0.1

**p<0.05

***p<0.01

**Table 13:**
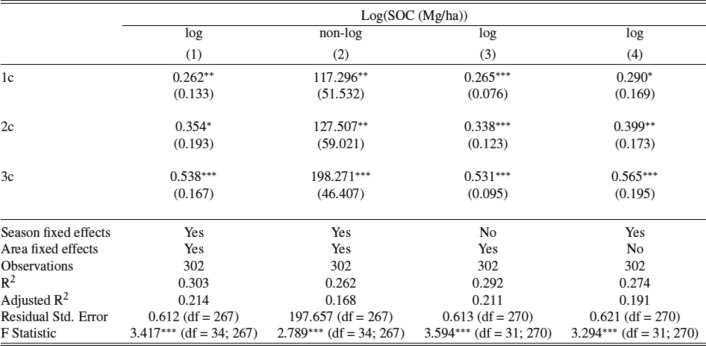
The effects of different sequences of corn-soybean rotation on log(SOC(Mg/ha C)) stocks across model specifications (raw regression result)

0c is base group. Clustered standard errors at the area level arew displayed in parantheses

*p<0.1

**p<0.05

***p<0.01

**Table 14:**
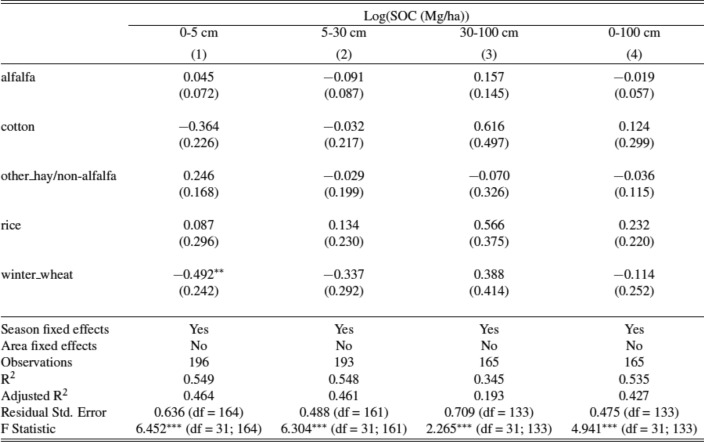
The effects of non-corn/soybean crops on log(SOC(Mg/ha C)) stocks without area fixed effects (raw regression result)

Spring wheat is base group. Clustered standard errors at the area level arew displayed in parantheses

*p<0.1

**p<0.05

***p<0.01

**Table 15:**
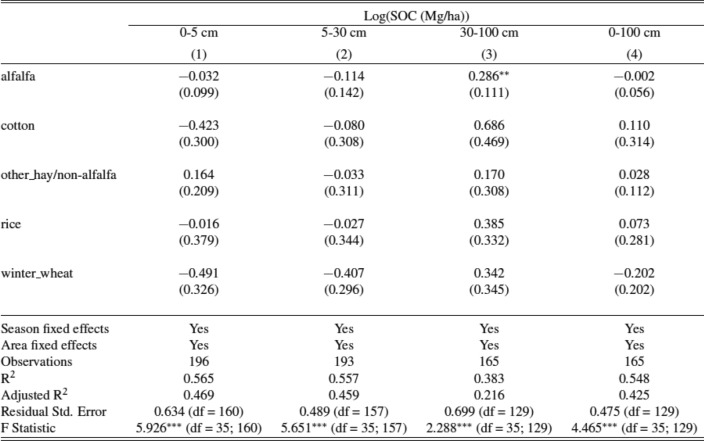
The effects of non-corn/soybean crops on log(SOC(Mg/ha C)) stocks with area fixed effects (raw regression result)

Spring wheat is base group. Clustered standard errors at the area level arew displayed in parantheses

*p<0.1

**p<0.05

***p<0.01

## Data Availability

The data that support the findings of this study were derived from the following resources available in the public domain: 1.RaCA dataset is available from https://www.nrcs.usda.gov/resources/data-and-reports/rapid-carbon-assessment-raca 2.CDL dataset can be downloaded from https://www.nass.usda.gov/Research_and_Science/Cropland/Release/index.php 3.PRISM dataset is accessed from https://prism.oregonstate.edu/

## Appendix

See Tables 6, 7, 8, 9, 10, 11, 12, 13, 14, 15 and 16 here.

**Table 6:** Descriptive Statistics

Variable			Mean	Sd	Min	Max	N
Soil Organic Carbon	0–5 cm		17.15	22.42	0.46	262.72	351
Stocks (Mg/ha C)	5–30 cm		68.84	110.94	2.78	1005.52	348
	30–100 cm		97.25	225.86	4.17	2076.73	320
Crop sequences	3c	ccc					181
(number of years of corn planted), (corn/soybean 2 years before—corn/soybean 1 year before—corn/soybean the year of measurement)	2c	ccs					41
		csc					287
		scc					69
	1c	css					35
		scs					304
		ssc					26
	0c	sss					76
Soil texture	clay						20
	clay loam						42
	fine sand						6
	fine sandy loam						46
	loam						102
	loamy fine sand						15
	loamy sand						17
	loamy very fine sand						3
	sand						6
	sandy clay loam						3
	silt						9
	silty clay						24
	silty clay loam						162
	silt loam						458
	sandy loam						28
	vary coarse sandy loam						24
Area	Midwest						770
	Northeast						72
	Southeast						159
	West						18
Year	2010						490
	2011						529
Season	spring	Mar-May					415
	summer	Jun-Aug					66
	fall	Sep-Nov					464
	winter	Dec-Feb					74
Latitude (degree)			40.34	2.85	32.81	47.34	1019
Mean temperature	past 1–6 warm months		20.26	2.46	14.03	25.87	1019
(degree Celsius)	past 1–6 cold months		2.02	3.75	-7.39	11.22	1019
	past 7–18 warm months		18.71	2.19	13.5	24.26	1019
	past 7–18 cold months		1.97	4.01	-8.1	11.91	1019
Mean precipitation	past 1–6 warm months		115.72	31.69	4.67	222.87	1019
(inch)	past 1–6 cold months		62.95	26.35	13.57	150.04	1019
	past 7–18 warm months		105.77	24.43	2.09	170.48	1019
	past 7–18 cold months		72.65	27.74	11.35	146.82	1019

**Table 16:** Area of land in various crop rotations 2019–2021 excluding 0.5 km buffer around open water, and potential change in SOC stocks across policies and states

	OH	IA	IL	IN	Corn Belt
Area of land (hectare)					
Mono-cropped with soybeans out of 3 years	211,878	16,396	99,372	108,239	435,886
Increase of SOC (Mg C)					
by adding 1 year of corn	6,963,243	538,857	3,265,794	3,557,214	14,325,109
by adding 2 years of corn	9,876,412	764,296	4,632,085	5,045,424	20,318,218
by adding 3 years of corn	16,567,635	1,282,103	7,770,301	8,463,675	34,083,714
Area of land (hectare)					
Cropped with soybeans for 1 year and corn for 2 years out of 3 years	923,848	2,279,466	2,493,299	1,187,567	6,884,179
Increase of SOC (Mg C)					
by adding 1 year of corn	12,814,722	31,618,552	34,584,632	16,472,781	95,490,688
by adding 2 years of corn	42,246,044	104,236,264	114,014,483	54,305,496	314,802,288
Area of land (hectare)					
cropped with soybeans for 2 years and corn for 1 year out of 3 years	578,167	3,265,437	2,832,663	1,099,136	7,775,403
Increase of SOC (Mg C)					
by adding 1 year of corn	20,510,192	115,839,857	100,487,408	38,991,338	275,828,795
